# Spatial variation and clustering of anaemia prevalence in school-aged children in Western Kenya

**DOI:** 10.1371/journal.pone.0282382

**Published:** 2023-11-27

**Authors:** Bibian N. Robert, Anitah Cherono, Eda Mumo, Charles Mwandawiro, Collins Okoyo, Paul M. Gichuki, Justine l. Blanford, Robert W. Snow, Emelda A. Okiro

**Affiliations:** 1 Kenya Medical Research Institute-Wellcome Trust Research Programme, Population and Health Impact Surveillance Group, Nairobi, Kenya; 2 Kenya Medical Research Institute, Eastern and Southern Africa Centre of International Parasite Control (ESACIPAC), Nairobi, Kenya; 3 Department of Epidemiology, Kenya Medical Research Institute, Statistics and Informatics (DESI), Nairobi, Kenya; 4 Department of Earth Observation Sciences, University of Twente, Enschede, Netherlands; 5 Nuffield Department of Medicine, Centre for Tropical Medicine and Global Health, University of Oxford, Oxford, United Kingdom; Washington State University, UNITED STATES

## Abstract

Anaemia surveillance has overlooked school-aged children (SAC), hence information on this age group is scarce. This study examined the spatial variation of anaemia prevalence among SAC (5–14 years) in western Kenya, a region associated with high malaria infection rates. A total of 8051 SAC were examined from 82 schools across eight counties in Western Kenya in February 2022. Haemoglobin (Hb) concentrations were assessed at the school and village level and anaemia defined as Hb<11.5g/dl for age 5-11yrs and Hb <12.0g/dl for 12-14yrs after adjusting for altitude. Moran’s I analysis was used to measure spatial autocorrelation, and local clusters of anaemia were mapped using spatial scan statistics and local indices of spatial association (LISA). The prevalence of anaemia among SAC was 27.8%. The spatial variation of anaemia was non-random, with Global Moran’s I 0.2 (p-value < 0.002). Two significant anaemia cluster windows were identified: Cluster 1 (LLR = 38.9, RR = 1.4, prevalence = 32.0%) and cluster 2 (LLR = 23.6, RR = 1.6, prevalence = 45.5%) at schools and cluster 1 (LLR = 41.3, RR = 1.4, prevalence = 33.3%) and cluster 2 (LLR = 24.5, RR = 1.6, prevalence = 36.8%) at villages. Additionally, LISA analysis identified ten school catchments as anaemia hotspots corresponding geographically to SatScan clusters. Anaemia in the SAC is a public health problem in the Western region of Kenya with some localised areas presenting greater risk relative to others. Increasing coverage of interventions, geographically targeting the prevention of anaemia in the SAC, including malaria, is required to alleviate the burden among children attending school in Western Kenya.

## Introduction

Anaemia is a global public health problem, affecting approximately 1.8 billion people worldwide in 2019, and is a leading cause of morbidity and mortality among pregnant women and young children [1,2]. Sub-Saharan Africa (SSA) bears the greatest burden of anaemia [2,3]. The World Health Organization (WHO) defines anaemia as a condition in which the number of red blood cells (oxygen-carrying capacity) is inadequate to meet the body’s physiological needs [4]. Iron deficiency is the main cause of anaemia. However, other factors leading to anaemia include malnutrition, inherited red blood cell disorders, infections from intestinal parasites, schistosomiasis, malaria, and human immunodeficiency virus (HIV) [5,6]. The risk factors associated with anaemia vary according to geography, age, and gender [1,7]. Therefore, it is important to understand the complex epidemiology of anaemia to successfully develop appropriate targeted, context-specific interventions [7,8].

However, population surveys and monitoring of anaemia have mainly focused on women of reproductive age (WRA) (15–49 years) and children under 5 years of age. This aligns with global targets for maternal, infant, and young child nutrition, which is committed to halve anaemia prevalence in WRA by 2025 [7]. Furthermore, the prevalence of anaemia in WRA was added as indicator 2.2.3 of the Sustainable Development Goals (SDGs) [9]. Despite the existence of important global health milestones, they overlook other vulnerable populations, particularly school-aged children (SAC) and the elderly (60+ years).

Owing to their rapid physical and physiological growth, SAC are more susceptible to anaemia [10–14]. There are limited national and global reports on the prevalence of anaemia among SAC [12,15,16], largely due to insufficient data in this age group [7,17]. Despite limited data, a review in 2008 suggested that as many as 64–71% of SAC could be anaemic in Africa [15]. SAC anaemia burden and its spatial patterns within countries remain poorly defined. Therefore, the purpose of this study was to determine the spatial distribution of anaemia at school level, among SAC in Western Kenya, specifically examining whether there were clusters of anaemia and identifying their location.

## Methodology

### Study context

**Fig 1:**
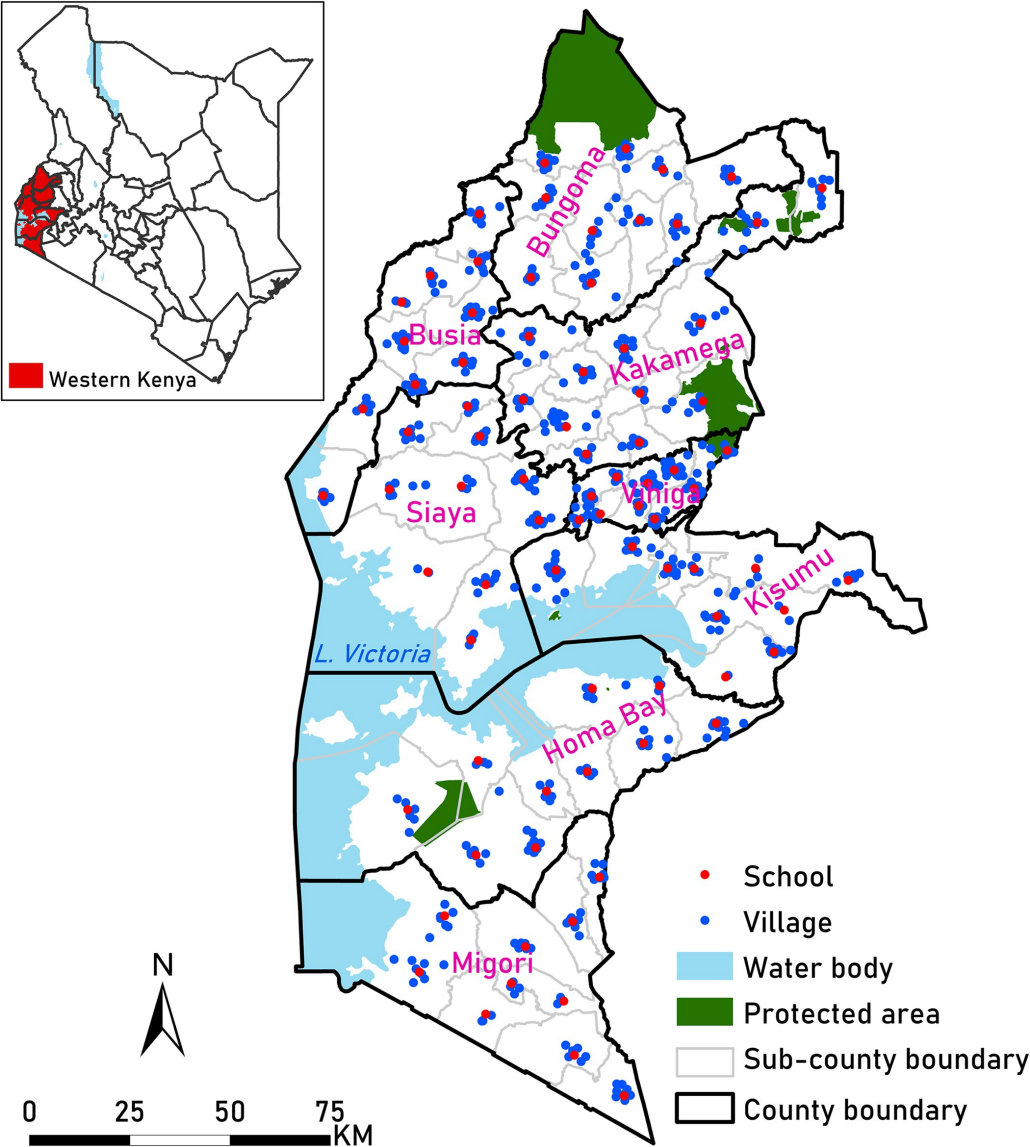
Study area map. Map of Western Kenya showing the distribution of schools and villages across the eight counties. The Western Kenya county and sub-county level shapefile was based on the County Integrated Development Plans 2021 [22].

This study was conducted across eight counties in Western Kenya: Bungoma, Busia, Kakamega, Vihiga, Siaya, Kisumu, Homa Bay and Migori. These counties represent the devolved system of governance in Kenya responsible for decision-making at the subnational level and are further divided into 62 sub-counties (Fig 1). This area, in the Lake Victoria Basin, is characterised by a high population density, representing 19.4% (9.4 million) of Kenya’s population [18]. The area was chosen because it experiences stable malaria transmission [19,20] and until recently was endemic to hookworms [21].

### Data

In February 2022, a survey was conducted on children attending 82 public primary schools in eight counties within a high malaria transmission region, where school surveys are frequently carried out. A sampling frame of all primary schools in the Western region was used to select the schools, and one school was randomly chosen from each sub-county in the eight selected counties (Fig 1). Approximately 100 children aged 5–14 years were randomly sampled from Classes 2 to 6 at each school. Selected children were asked to provide a finger-prick blood sample, which was used to assess their haemoglobin concentration using a portable photometer (HemoCue AB, Ängelholm, Sweden). The location of each sampled school was recorded using a hand-held Global Positioning System (GPS). The children provided information on their village of residence with the help of their teachers. Village coordinates were geolocated using Google Earth/Maps and digital place-name gazetteers, including Geonames, Encarta, and OpenStreetMap. The village locations were validated in ArcMap version 10.5 (ESRI Inc., Redlands, CA, USA) using the approximate travel time to schools reported by the children.

Altitude was extracted from the shuttle radar topographic mission digital elevation models (SRTM) at the 30m x 30m spatial resolution downloaded from Regional Centre for Mapping of Resources for Development [23]. Data on protected areas were based on Biodiversity and Protected Areas Management Reference Information System (RIS) [24] and waterbodies downloaded from the Humanitarian Data Exchange website [25].

### Anaemia prevalence

Hb measurements were adjusted for altitude according to the Centers for Disease Control and Prevention recommendations (Hb=−0.32×[altitudeinmetres×0.0033]+0.22×[altitudeinmetres×0.0033]) [26]. No adjustment was made for altitudes below 1000 m. Anaemia was defined using the following age-specific thresholds: <11 g/dl for children aged <5 years, <11.5 g/dl for children aged 5–11 years, and <12 g/dl for children aged 12–14 years. The prevalence of anaemia at school was calculated as the proportion of children with anaemia out of those sampled in each school. A test of proportions was conducted to evaluate whether there was a significant difference between the proportion of children with anaemia by gender. The prevalence value from the school was then adopted to the school catchment area, derived using spatial techniques explained in the next section (School catchment area). Anaemia prevalence was also estimated at the village-level address, to assess clusters based on the area of residence.

### School catchment area

The school catchment area, a geographical area around a school, was defined as the adjacent area representing most SAC that attended that school. The schools were mapped to the villages of each child (S1 Fig) using the children’s geolocated residential addresses and the catchment was delineated by creating Thiessen Polygons in ArcMap V.10.5. This approach divides the area covered by point features (schools) into proximal zones such that any location within the zone is closer to its associated school than to any other school within the study area [27–29].

### Spatial analysis

The geographical patterns of anaemia prevalence were assessed within the study area using local and global spatial autocorrelation measures to identify hotspots and outliers. First, the existence of clusters was determined using Global Moran’s I spatial statistic, where values range from − 1 (negative spatial association) to + 1 (event\disease is clustered), with 0 indicating no spatial autocorrelation (uniform distribution of disease\event). A (p < 0.05) was used to define significant spatial autocorrelation.

Next, the locations of the clusters were identified using a circular spatial window. The presence of statistically significant spatial hotspots/clusters of anaemia was tested using a Bernoulli model using Kuldorff’s SaTScan (v.10.0.2) software. SACs with anaemia at each school location were considered cases and those without were considered controls to fit the Bernoulli model. The default maximum spatial cluster size of <50% of the at-risk population was used [30]. The significance of each potential cluster was evaluated using a likelihood ratio test statistic, and a p-value of 0.05 was considered significant. The scanning window with the maximum likelihood was the most likely cluster (primary cluster), and a p-value was assigned based on Monte Carlo hypothesis testing. Secondary clusters were only reported if they did not overlap with a primary cluster or a previously reported cluster [30]. Cluster analysis was repeated at the village to assess differences in spatial patterns in areas of residence where risk factors of the disease are and at school sampling sites.

Cluster detection was also undertaken at the areal level (school catchments) to identify anaemia hotspots across catchment areas and as a sensitivity analysis, given the assumption that the prevalence estimated at school is represented in the school catchment. Geoda v 1.20.0.10, was used to generate Anselin’s local indicator of spatial association (LISA) test statistic that identifies neighbouring geographical units (catchments) with statistically similar disease rates [31]. The statistical significance level for each spatial unit was computed and mapped onto a LISA significance map.

The distribution of anaemia prevalence across counties and within the identified SatScan and LISA clusters was summarised using box plots. In addition, the Kruskal-Wallis rank sum test was employed to assess whether there were significant differences in anaemia prevalence values between schools and villages and LISA clusters.

### Software

SaTScan™ software (v. 10.0.2, Kulldorff and Information Management Services, Inc.) and Geoda software (v. 1.20.0.10) were used for the cluster analysis. ArcMap (v10.5; ESRI Inc., Redlands, CA, USA) was used for geoprocessing and mapping. Data analysis and management were performed using R software (Version 4.2) and StataCorp. 2021 [Stata Statistical Software, Release 17. College Station, TX: StataCorp LP]

## Ethical approval and consent to participate

Ethical review approval was obtained from the Kenya Medical Research Institute Scientific Ethics Review Unit SERU (#2801; #3822; #3832), Ministry of Education (MOE/ECD/VoII/37/35), and Ministry of Health (MOH/ADM/1/1/2). At the school level, parental consent was based on passive, opt-out consent rather than written opt-in consent owing to the low risk and routine nature of the study procedures. Individual assent was obtained from each child before participating in the survey. The data analysed here did not include individual identification variables hence confidentiality was maintained.

## Results

### Socio-demographic characteristics

A total of 8085 children were sampled across 82 schools:4030 females and 4055 males. Each county had ten sampled schools, except for Kakamega, where 12 schools were sampled (Fig 1). Most (76%) of the children sampled were between 5 and 11 years of age. Data on Hb concentration were available for 8051 (99.6%) children out of 8085 who were sampled. Children with missing Hb concentration data were excluded from the analysis. Seventy-five percent of the villages were geocoded and included in the analysis (Fig 1).

### Anaemia prevalence among SAC

**Fig 2:**
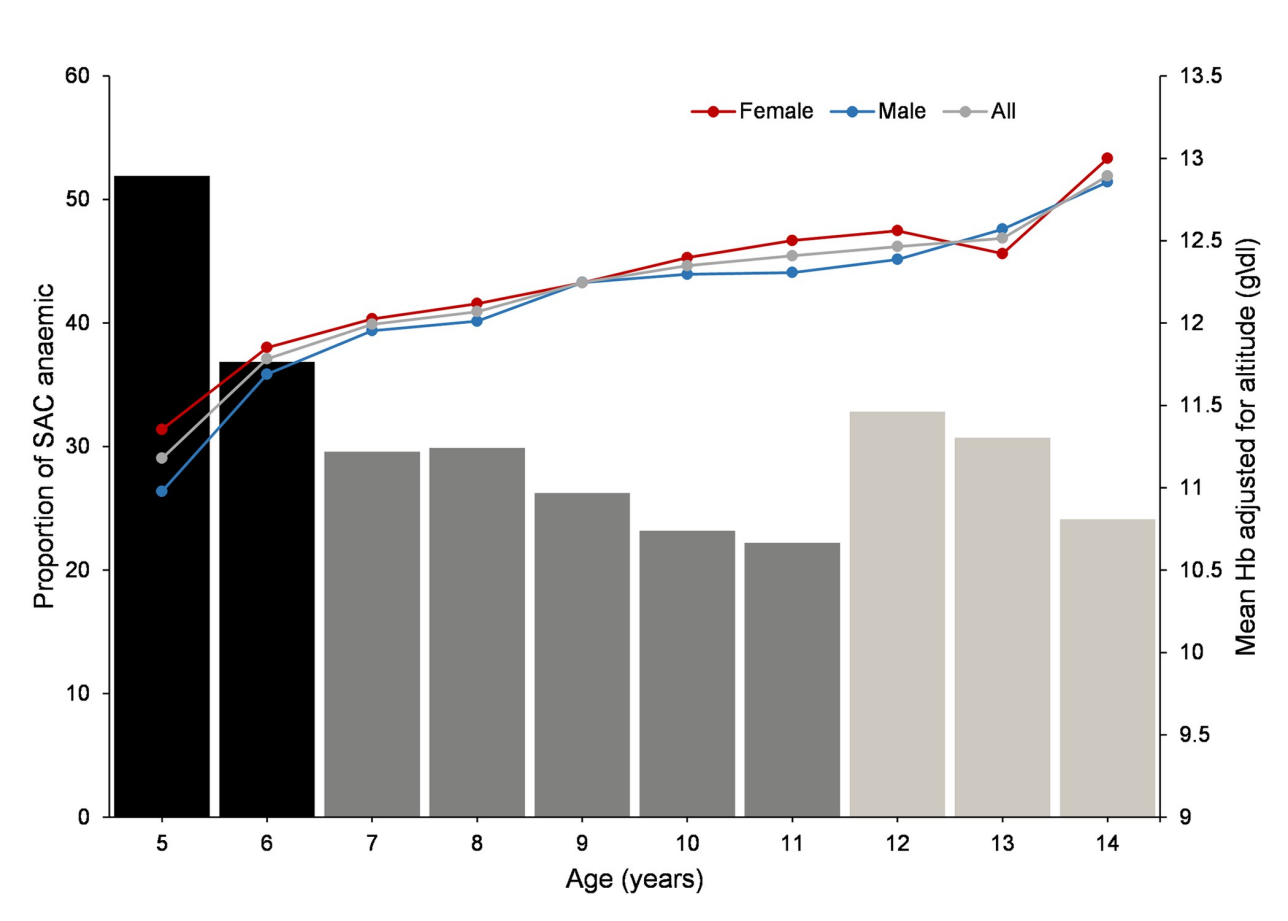
Combined bar and line charts. The bar chart shows the variation in anaemia prevalence by age (5–14 yrs) and the line graph shows mean Hb (adjusted for altitude) by gender across all age groups (5–14 yrs).

**Fig 3:**
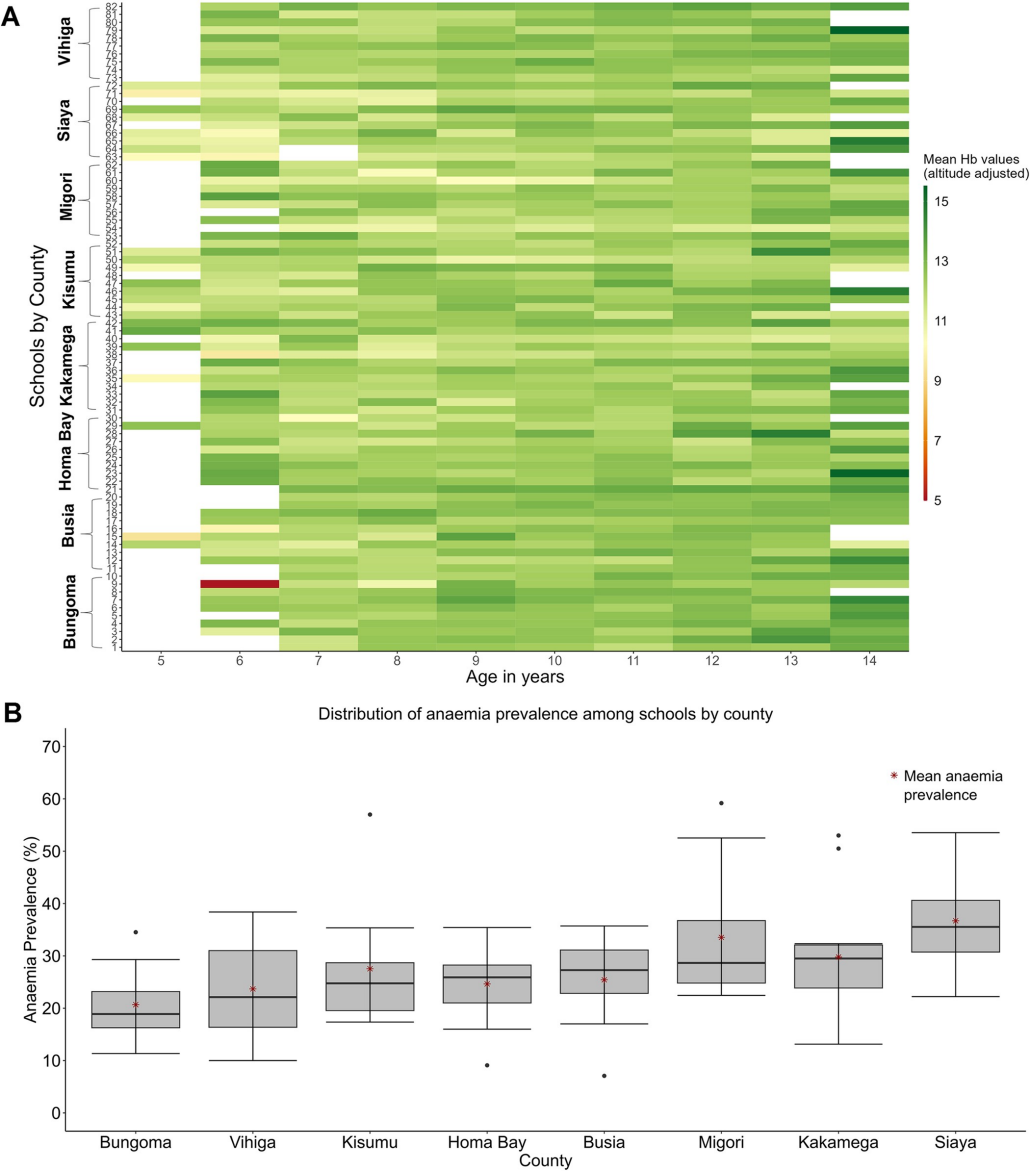
(A) Variation of mean Hb (adjusted for altitude) for each age (5–14 yrs) across the 82 schools. The blanks in white indicate missing samples for that age group. (B) Boxplots showing the distribution of anaemia prevalence among schools by county.

Overall, 27.8% of SAC were classified as anaemic. There was no statistically significant difference in anaemia prevalence between males (28.5%) and females (27.2%) (chi = 1.5913, p = 0.207). The prevalence of anaemia varied with age (Fig 2). The 5 to 6 years age group had the highest prevalence at 40.8% (Fig 2), and the adjusted Hb concentration among children ranged between 2.0 to 19.6 g/dl, with a mean value of 12.3 g/dl (95% CI:26.8–28.8%) (Table 1). The mean Hb increased steadily with age; however, there was a slight drop (12.6 g/dl to 12.4 g/dl) in females between ages 12 and 13 (Fig 2). There was minimal variation in mean Hb by age across schools as shown in Fig 3A.

**Table 1:** Summary statistics of Hb concentration (g/dl) by age category and sex across counties.

County	Gender	Age Categories	Hb Concentration (Adjusted for altitude)					
			min	max	sd	mean	median	n
**Bungoma**	Female	12–14 yrs	7.7	15.4	1.3	12.9	12.9	106
		5–11 yrs	5.1	16.6	1.4	12.6	12.7	368
	Male	12–14 yrs	7.0	16.3	1.4	12.8	12.8	160
		5–11 yrs	7.2	16.2	1.5	12.4	12.5	319
	**Total**	**5–14 yrs**	**5.1**	**16.6**	**1.4**	**12.6**	**12.7**	**953**
**Busia**	Female	12–14 yrs	9.7	16.7	1.4	12.8	12.9	111
		5–11 yrs	7.7	16.8	1.4	12.3	12.3	382
	Male	12–14 yrs	8.8	16.4	1.3	12.7	12.8	160
		5–11 yrs	8.2	15.7	1.3	12.4	12.5	335
	**Total**	**5–14 yrs**	**7.7**	**16.8**	**1.3**	**12.5**	**12.5**	**988**
**Homa Bay**	Female	12–14 yrs	6.7	15.5	1.5	12.4	12.5	100
		5–11 yrs	2.0	15.9	1.5	12.4	12.5	387
	Male	12–14 yrs	7.8	15.6	1.5	12.7	12.8	135
		5–11 yrs	4.8	16.3	1.5	12.3	12.4	357
	**Total**	**5–14 yrs**	**2.0**	**16.3**	**1.5**	**12.4**	**12.5**	**979**
**Kakamega**	Female	12–14 yrs	7.5	16.7	1.6	12.3	12.2	101
		5–11 yrs	6.4	16.1	1.4	12.2	12.2	494
	Male	12–14 yrs	5.5	15.1	1.4	12.3	12.4	174
		5–11 yrs	5.2	19.5	1.6	12.0	12.2	426
	**Total**	**5–14 yrs**	**5.2**	**19.5**	**1.5**	**12.2**	**12.3**	**1195**
**Kisumu**	Female	12–14 yrs	6.2	16.5	1.5	12.3	12.1	86
		5–11 yrs	4.0	16.0	1.5	12.2	12.3	397
	Male	12–14 yrs	7.9	15.3	1.4	12.6	12.8	121
		5–11 yrs	5.3	16.0	1.4	12.1	12.2	363
	**Total**	5–14 yrs	**4.0**	**16.5**	**1.4**	**12.2**	**12.3**	**967**
**Migori**	Female	12–14 yrs	6.4	18.0	1.6	12.5	12.6	106
		5–11 yrs	3.1	16.3	1.8	12.0	12.3	387
	Male	12–14 yrs	3.2	16.2	1.8	12.2	12.3	151
		5–11 yrs	4.6	19.6	1.7	11.9	12.1	337
	**Total**	**5–14 yrs**	**3.1**	**19.6**	**1.7**	**12.1**	**12.2**	**981**
**Siaya**	Female	12–14 yrs	8.6	15.5	1.5	12.5	12.9	111
		5–11 yrs	6.1	17.0	1.6	12.0	12.1	382
	Male	12–14 yrs	5.1	16.6	1.7	12.3	12.5	151
		5–11 yrs	6.3	16.1	1.6	11.8	11.9	348
	**Total**	**5–14 yrs**	**5.1**	**17.0**	**1.7**	**12.0**	**12.1**	**992**
**Vihiga**	Female	12–14 yrs	10.5	15.7	1.3	12.8	12.9	69
		5–11 yrs	8.0	16.8	1.3	12.4	12.5	429
	Male	12–14 yrs	7.4	15.2	1.4	12.5	12.6	112
		5–11 yrs	4.9	16.5	1.5	12.3	12.4	386
	**Total**	**5–14 yrs**	**4.9**	**16.8**	**1.4**	**12.4**	**12.5**	**996**
**Western Region**	Female	12–14 yrs	6.2	18.0	1.5	12.6	12.6	790
		5–11 yrs	2.0	17.0	1.5	12.3	12.4	3226
	Male	12–14 yrs	3.2	16.6	1.5	12.5	12.6	1164
		5–11 yrs	4.6	19.6	1.5	12.2	12.3	2871
	**Total**	**5–14 yrs**	**2.0**	**19.6**	**1.5**	**12.3**	**12.4**	**8051**

### Spatial distribution of anaemia prevalence

**Fig 4:**
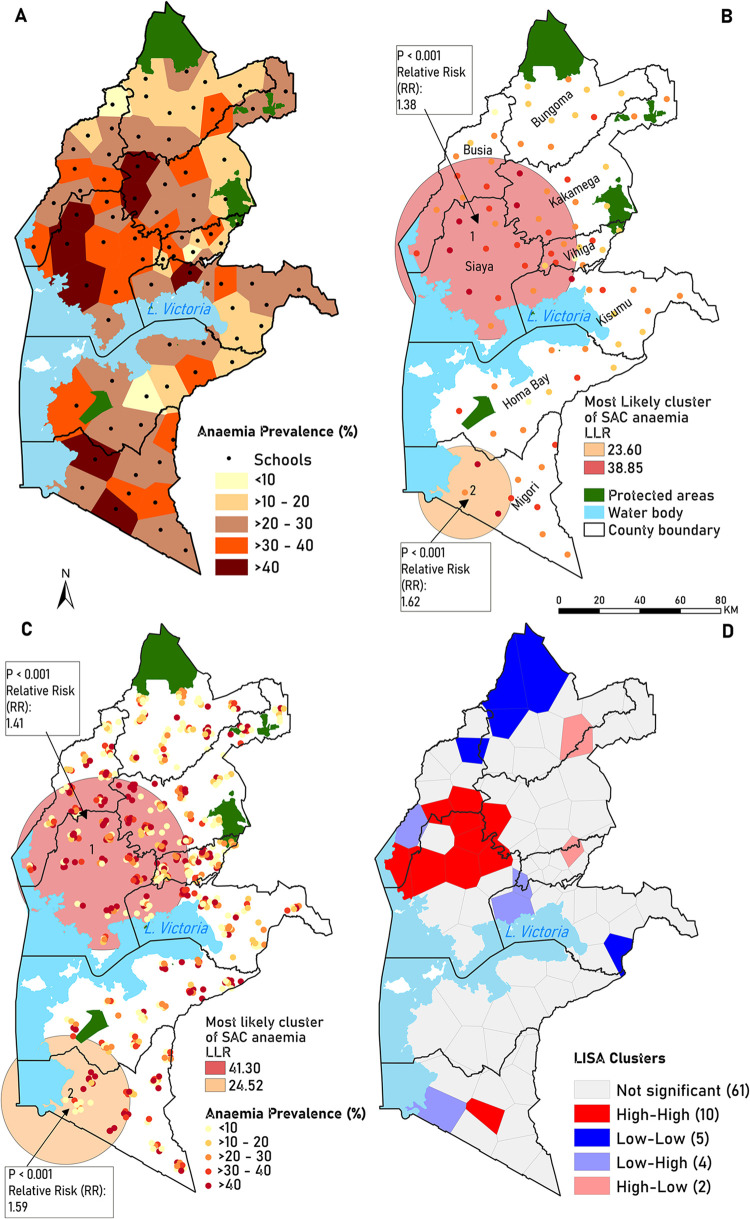
(A) Anaemia prevalence for each school catchment (computed from empirical Hb measurements), (B) Spatial scan statistics results of anaemia clusters for schools, (C) Spatial scan statistics results of anaemia clusters for villages and (D) LISA cluster map showing anaemia hotspots (red) and cold spots (blue). The corresponding LISA significance map in shown in S2 Fig. The Western Kenya county level shapefile was based on County Integrated Development Plans 2021 [22].

The empirical prevalence of anaemia varied across schools (Fig 3B), ranging from 7.1% to 59.2%. The prevalence value at each school was adopted for the corresponding school catchment areas (Fig 4A). Counties with the highest (>40%) prevalence of school catchment anaemia included Kakamega, Siaya, Migori, and Kisumu. At the county level, Siaya had the highest mean prevalence of anaemia (36.7%) and Bungoma had the lowest (20.6%). Siaya, Migori and Vihiga showed the greatest variability in prevalence values across schools compared to other counties with median prevalence values of 35.5% (30.7–40.6), 27.8% (24.8–36.7) and 22.1% (16.4–31.0) respectively (Fig 3B). In contrast, the distribution of anaemia prevalence in Bungoma 18.9% (16.3–23.2), Busia 27.3% (22.8–31.1), Homa Bay 25.9% (21.0–28.2) and Kisumu 24.8% (19.6–28.7) appeared to be relatively symmetrical. However, the distribution of anaemia prevalence in Kakamega skewed towards lower prevalence values of 29.5% (23.9–32.1), while Migori displayed skewness towards higher prevalence values.

### Spatial autocorrelation and clustering

The Global Moran’s I value of 0.2 (p <0.002) indicated significant clustering of anaemia within the study area. Spatial SatScan statistics identified two significant cluster spatial windows: primary cluster 1 (939 out of 769 expected cases) traversing Siaya County and parts of Busia, Kakamega, Vihiga, and Kisumu counties bordering Siaya County, and secondary cluster 2 (171 out of 109 expected cases) located in the southern part of the study area, covering a section of Migori County (Fig 4B). Cluster 1 had the highest Log-Likelihood ratio (LLR) of 38.9 with a relative risk (RR) of 1.4 implying that children within this cluster are at 40% more risk than those residing outside. In contrast, cluster 2 had an LLR of 23.6 with an RR of 1.6 and a 60% increased risk of anaemia. A total of 28 schools were within Cluster 1, and the majority (36%) were in Siaya County. Only four schools comprised cluster 2. Cluster analysis at the village level (location of residence) highlighted spatial patterns similar to those at schools (LLR = 41.3, RR = 1.4) for Cluster 1 and (LLR = 24.5, RR = 1.6) for Cluster 2 (Fig 4C).

The sensitivity analysis of the LISA results of the areal level clustering (at school catchments) are shown in (Fig 4D). A total of 10 catchments constituted the hotspots (high-high) shown in red, implying that catchments with high anaemia prevalence are surrounded by neighbouring catchments with high anaemia prevalence. In contrast, five cold spots (low-low) in blue indicate the opposite: a region of low anaemia prevalence. Six of the clusters indicated discordance with either low prevalence surrounded by high prevalence (low-high clusters), or high prevalence surrounded by low prevalence (high-low clusters). The ten hotspots (clusters with high anaemia prevalence) were located in the western part, north of Lake Victoria, and a section in the southern part of the study area. These clusters correspond spatially to those identified by the spatial scan statistical analysis (Fig 4B), and catchments were estimated to have a high prevalence of anaemia (Fig 4A).

**Fig 5:**
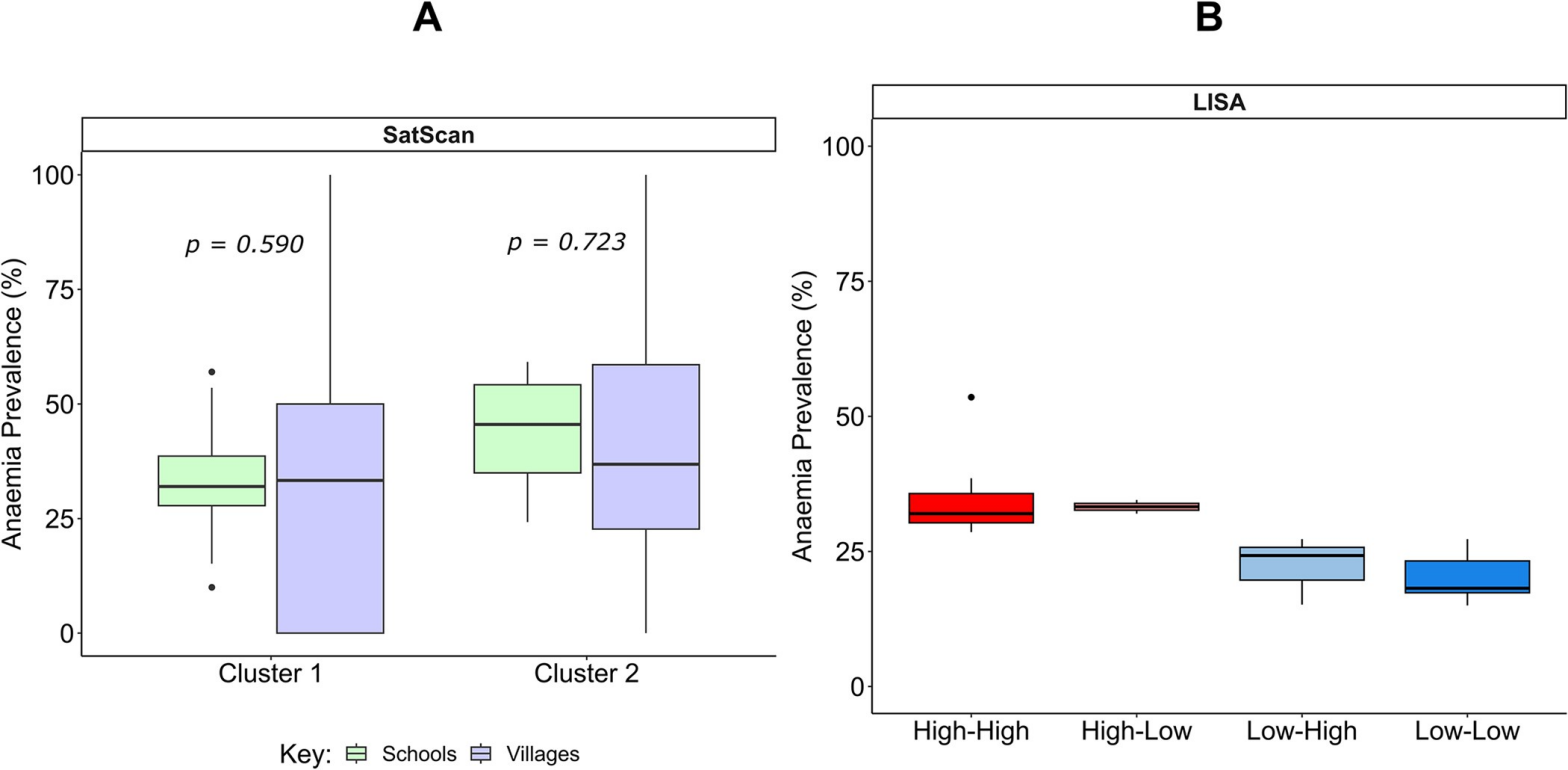
Box plot of the prevalence rates across clusters identified by SatScan and LISA cluster analysis. (A) shows distribution of anaemia prevalence across schools and villages in cluster 1 and cluster 2 identified from SatScan analysis; (B) shows distribution of anaemia prevalence across High-High, High-Low, Low-High and Low-Low LISA clusters.

Fig 5A illustrates the distribution of anaemia prevalence across schools and villages by SatScan clusters. In cluster 1, the median anaemia prevalence in schools is 32.0% (27.8–38.6) and is 33.3% (0–50) in villages. However, the observed variation between schools and villages was not statistically significant (chi = 0.2904, p = 0.590). Similarly, in cluster 2, the median prevalence was 45.5% (35.0–54.2) in schools and 36.8% (22.7–58.6) in villages, with no statistically significant variation found (chi = 0.1257, p = 0.723).

Across the four LISA clusters, the high clusters (High-High and High-Low) clusters had the highest median anaemia prevalence, with values of 32.0% (30.3–35.7) and 33.3% (32.6–33.9) respectively. In contrast, the Low clusters (Low-High and Low-Low) had lower median anaemia prevalence: 24.2% (19.7–25.8) for the Low-High cluster and 18.2% (17.4–23.2) for the Low-Low cluster (Fig 5B). The variation between the high and low clusters was statistically significant (chi = 13.235, p < 0.05).

## Discussion

The government of Kenya recognizes that improving the health of learners is a critical driver for achieving Kenya Vision 2030 [32]. Unfortunately, little is known about the prevalence of anaemia among SAC, although this condition contributes to poor intellectual and physical development among this population [13,17]. In this study, we employed global and local spatial autocorrelation techniques to assess the geographical distribution of anaemia prevalence in SAC in Western Kenya. Over 25% of the SAC were anaemic, ranging from 20.6% in Bungoma to 36.7% in Siaya. Our findings underline the spatial heterogeneity of anaemia prevalence in this area of Kenya.

Overall, the prevalence of SAC anaemia in Western Kenya was 27.8%. Other similar studies conducted in Kenya reported the prevalence of SAC anaemia to be 35% (5–16 yrs) [33] and 28.8% (6 months -14 yrs) [34] in 2013 and 2014, respectively. In our study, the average Hb concentration increased with age, but anaemia prevalence decreased progressively with age within each age group (5-11yrs & 12-14yrs) (Fig 2), which is comparable to a previous study [34]. Across studies, there are disparities in the variation in Hb concentration and prevalence of anaemia by sex [35]. Other studies in Kenya suggest that male SAC are at a higher risk of anaemia than female SAC [17,34]. The difference in this study was not statistically significant, which is consistent with earlier studies conducted in Ethiopia [16,36]. However, we have limited knowledge of what constitutes a biological framework for anaemia in SAC.

Spatial autocorrelation analysis revealed a non-random distribution of SAC anaemia. Although the spatial scan statistics identified more geographical locations (32) as high-risk anaemia areas compared to LISA (10), there was spatial consistency in the locations of these hotspots [37]. Generally, children residing in the western part of the study area, north of Lake Victoria, and southern parts of Migori County are at an elevated risk of anaemia compared to children residing in other regions of the study area. Based on spatial scan statistics, no significant differences in median anaemia prevalence were observed at the village and schools within cluster 1 and cluster 2. This finding suggests that the prevalence of anaemia in schools in Western Kenya represent where the children live and reflects the contextual residential characteristics [38]. However, there was a significant difference in median anaemia prevalence between high and low clusters from LISA analysis, further highlighting the spatial disparity of anaemia prevalence across school catchments. These results from the cluster analysis are useful for informing a targeted approach and prioritising resources as has been used in targeting malaria interventions [39]. Further, these findings form a basis for conducting further studies to understand the underlying factors of anaemia better. Specifically, SatScan provides a general overview of where clusters (Fig 4B and 4C) are, whereas LISA provides insights into which school catchments should be prioritised (Fig 4D).

In addition to hot spot areas, LISA identified outlier areas (areas with high anaemia prevalence close to areas with low anaemia prevalence and where areas with high prevalence surround high prevalence areas) (Fig 4D) [40]. These results provide additional insights for developing tailored strategies and policies considering spatial dependencies and relationships between neighbouring regions [41].

A complex configuration of causes such as socioeconomic status, environmental factors, food security, and differences in the prevalence of parasitic infections, such as soil-transmitted helminth infection, hookworms, and malaria, may contribute to anaemia risk [42–45]. Food insecurity for example, can cause nutritional deficiencies which are linked with anaemia [46]. However, in Western Kenya, where over 80% of the population is food secure according to the FEWS NET Integrated Phase Classification [47], food security is unlikely to be a significant driver of anaemia; instead, the region’s high malaria transmission rates may be a key contributing risk factor for anaemia [48], as are variation in access to clean water.

The prevalence of anaemia can be reduced through a variety of interventions. In Kenya, some of the indirect interventions to manage parasitic infection for example, deworming to reduce the burden of intestinal parasites, promoting good hygiene, and promoting malaria intervention [49–52] have been implemented through National School-Based Deworming Programme (NSBDP) and the National Malaria Control Programme (NMCP) [51,52]. Other direct interventions include those related to nutrition (e.g., promoting healthy diets, food fortification and supplementing diets such as iron, and folic acid) [53–55]. Cluster analysis results such as those presented here can assist in efficiently targeting these interventions and in better allocating limited resources complemented by further research to better understand the role of different factors on anaemia prevalence.

This study had several limitations. First, we assumed that the school sample represented a community. However, the subset of children who go to school may be different from those who do not, but there is no information to assess this. Second, more than 50% of schools lacked samples of 5 years old children (Fig 3A), which could potentially underestimate anaemia prevalence at these schools because younger children are more vulnerable to anaemia. Third, the choice of the cluster window size is subjective. However, this study used a recommended window size of 50% of the study population to detect both small and large clusters [30]. Fourth, though the use of circular scan windows may hinder the detection of irregular clusters, they are rotationally invariant and provide clear signals of disease hotspots for public health decision-makers and stakeholders [56–60]. Lastly, it is important to highlight that our study specifically focused on the clustering of anaemia and did not include an analysis of associated risk factors. Although extensive research has been conducted on these factors in various contexts, including Kenya, providing a valuable resource for future investigations [8,34,38,44], the results from this study are useful for directing where further research should be conducted to better understand the factors driving anaemia within specific geographic locations since variation within school catchments can occur.

## Conclusion

The prevalence of anaemia among SAC in Western Kenya is a moderate public health concern, as determined by WHO thresholds, with a prevalence of 27.8% [4,7]. Our findings indicate a need to expand control measures to address anaemia in this population, and that further research is needed to better assess the underlying causes. In light of geographical differences in anaemia prevalence, it is crucial to allocate resources to hotspot areas to effectively reduce the burden of anaemia among SAC in Western Kenya. Effective interventions such as providing iron and folic acid supplements, promoting healthy diets and food fortification, deworming to reduce the burden of intestinal parasites, promoting good hygiene, and promoting malaria intervention should be prioritized to improve SAC health outcomes [51–54,60]. These interventions can be implemented through school feeding programs and other community- and school-based health intervention approaches [49–51].

## Supporting information

S1 Fig Thiessen polygons representing 82 school catchment areas. The Western Kenya shapefile was based on the County Integrated Development Plans 2021 [22].
(TIF)

S2 Fig LISA significance map. The Western Kenya county level shapefile was based on the County Integrated Development Plans 2021 [22].
(TIF)

S1 File (DOCX)

## References

[1] Kassebaum NJ, Jasrasaria R, Naghavi M, Wulf SK, Johns N, Lozano R, et al. A systematic analysis of global anemia burden from 1990 to 2010. Blood, the Journal of the American Society of Hematology. 2014;123(5):615–24. doi: 10.1182/blood-2013-06-508325 24297872 PMC3907750

[2] Safiri S, Kolahi A-A, Noori M, Nejadghaderi SA, Karamzad N, Bragazzi NL, et al. Burden of anemia and its underlying causes in 204 countries and territories, 1990–2019: results from the Global Burden of Disease Study 2019. Journal of hematology & oncology. 2021;14(1):1–16. doi: 10.1186/s13045-021-01202-2 34736513 PMC8567696

[3] WHO. Global Anaemia Estimates 2021 [cited 19^th^ December 2022]. Available from: https://www.who.int/data/gho/data/themes/topics/anaemia_in_women_and_children.

[4] WHO. Haemoglobin concentrations for the diagnosis of anaemia and assessment of severity. World Health Organization, 2011.

[5] Shaw JG, Friedman JF. Iron deficiency anemia: focus on infectious diseases in lesser developed countries. Anemia. 2011;2011. doi: 10.1155/2011/260380 21738863 PMC3124144

[6] WHO CDC. Assessing the iron status of populations: including literature reviews: report of a Joint World Health Organization/Centers for Disease Control and Prevention Technical Consultation on the Assessment of Iron Status at the Population Level, Geneva, Switzerland, 6–8 April 2004. 2nd ed. Geneva: World Health Organization; 2007.

[7] WHO. Global anaemia reduction efforts among women of reproductive age: impact, achievement of targets and the way forward for optimizing efforts. 2020.

[8] Chaparro CM, Suchdev PS. Anemia epidemiology, pathophysiology, and etiology in low‐and middle‐income countries. Annals of the new York Academy of Sciences. 2019;1450(1):15–31. doi: 10.1111/nyas.14092 31008520 PMC6697587

[9] UN. Transforming our world: the 2030 Agenda for Sustainable Development. 2015 [cited 20^th^ December 2022]. Available from: https://sdgs.un.org/2030agenda.

[10] Pasricha S-R, Drakesmith H, Black J, Hipgrave D, Biggs B-A. Control of iron deficiency anemia in low-and middle-income countries. Blood, The Journal of the American Society of Hematology. 2013;121(14):2607–17. doi: 10.1182/blood-2012-09-453522 23355536

[11] Tesfaye M, Yemane T, Adisu W, Asres Y, Gedefaw L. Anemia and iron deficiency among school adolescents: burden, severity, and determinant factors in southwest Ethiopia. Adolesc Health Med Ther. 2015;6:189–96 doi: 10.2147/AHMT.S94865 26719736 PMC4687608

[12] Brooker S, Clements AC, Bundy DA. Global epidemiology, ecology and control of soil-transmitted helminth infections. Advances in parasitology. 2006;62:221–61. doi: 10.1016/S0065-308X(05)62007-6 16647972 PMC1976253

[13] Lozoff B, Beard J, Connor J, Felt B, Georgieff M, Schallert T. Long-lasting neural and behavioral effects of iron deficiency in infancy. Nutrition reviews. 2006;64(suppl_2):S34–S43. doi: 10.1301/nr.2006.may.s34-s43 16770951 PMC1540447

[14] Szajewska H, Ruszczynski M, Chmielewska A. Effects of iron supplementation in nonanemic pregnant women, infants, and young children on the mental performance and psychomotor development of children: a systematic review of randomized controlled trials. The American journal of clinical nutrition. 2010;91(6):1684–90. doi: 10.3945/ajcn.2010.29191 20410098

[15] De Benoist B, Cogswell M, Egli I, McLean E. Worldwide prevalence of anaemia 1993–2005; WHO global database of anaemia. 2008. 10.1017/S1368980008002401 18498676

[16] Tezera R, Sahile Z, Yilma D, Misganaw E, Mulu E. Prevalence of anemia among school-age children in Ethiopia: a systematic review and meta-analysis. Systematic reviews. 2018;7(1):1–7. 29793528 10.1186/s13643-018-0741-6 PMC5968474

[17] Okiro EA, Joseph NK, Gitonga CW, Snow RW. Anaemia among Kenyan children: a call for improved monitoring and intervention in school-aged children. Transactions of the Royal Society of Tropical Medicine and Hygiene. 2020;114(8):627–31. doi: 10.1093/trstmh/traa032 32484872 PMC7405173

[18] KNBS. 2019 Kenya population and housing census, vol I: population by county and sub-county. Nairobi, Kenya 2019 [cited June 2022]. Available from: https://www.knbs.or.ke/?wpdmpro=2019-kenya-population-and-housing-census-volume-i-population-by-county-and-sub-county.

[19] Alegana VA, Macharia PM, Muchiri S, Mumo E, Oyugi E, Kamau A, et al. Plasmodium falciparum parasite prevalence in East Africa: updating data for malaria stratification. PLOS global public health. 2021;1(12):e0000014. doi: 10.1371/journal.pgph.0000014 35211700 PMC7612417

[20] Macharia PM, Odera PA, Snow RW, Noor AM. Spatial models for the rational allocation of routinely distributed bed nets to public health facilities in Western Kenya. Malaria Journal. 2017;16(1):367. doi: 10.1186/s12936-017-2009-3 28899379 PMC5596856

[21] Okoyo C, Campbell SJ, Williams K, Simiyu E, Owaga C, Mwandawiro C. Prevalence, intensity and associated risk factors of soil-transmitted helminth and schistosome infections in Kenya: Impact assessment after five rounds of mass drug administration in Kenya. PLoS Negl Trop Dis. 2020;14(10):e0008604. doi: 10.1371/journal.pntd.0008604 33027264 PMC7540847

[22] Figshare. Sub-counties of Kenya based on County Intergrated Development Plans 2021. CC BY 4.0. 10.6084/m9.figshare.12501455.v1.

[23] RCMRD. Kenya Srtm Dem 30meters [cited July 2022]. Available from: https://www.rcmrd.org/.

[24] BIOPAMA RIS. Biodiversity and Protected Areas Management Reference Information System [cited September 2023]. Available from: https://rris.biopama.org/ct/country/KE.

[25] HDX. Humanitarian Data Exchange 2022 [cited July 2022]. Available from: https://data.humdata.org/dataset.

[26] Sullivan KM, Mei Z, Grummer‐Strawn L, Parvanta I. Haemoglobin adjustments to define anaemia. Tropical Medicine & International Health. 2008;13(10):1267–71. doi: 10.1111/j.1365-3156.2008.02143.x 18721184

[27] Esri. GIS Dictionary 2021 [cited 5^th^ December 2022]. Available from: https://support.esri.com/en-us/gis-dictionary.

[28] Macharia PM, Odhiambo JN, Mumo E, Maina A, Giorgi E, Okiro EA. Approaches to defining health facility catchment areas in sub-Saharan Africa. medRxiv. 2022.

[29] Pokojski W, Pokojska P. Voronoi diagrams–inventor, method, applications. Polish Cartographical Review. 2018;50(3):141–50.

[30] Kulldorff M. SaTScanTM user guide for version 10.0. 2021.

[31] Anselin L, Syabri I, Kho Y. GeoDa: an introduction to spatial data analysis. Handbook of applied spatial analysis: Springer; 2010. p. 73–89.

[32] MOE, MOH. Kenya School Health Policy. 2018 [cited 29^th^ December 2022]. Available from:https://www.ncikenya.or.ke/documents/KENYA%20SCHOOL%20HEALTH%20POLICY%20BOOK%20_20_11_2018.pdf.

[33] Pullan RL, Gitonga C, Mwandawiro C, Snow RW, Brooker SJ. Estimating the relative contribution of parasitic infections and nutrition for anaemia among school-aged children in Kenya: a subnational geostatistical analysis. BMJ open. 2013;3(2):e001936. doi: 10.1136/bmjopen-2012-001936 23435794 PMC3586185

[34] Ngesa O, Mwambi H. Prevalence and risk factors of anaemia among children aged between 6 months and 14 years in Kenya. PLoS One. 2014;9(11):e113756. doi: 10.1371/journal.pone.0113756 25423084 PMC4244137

[35] Jorgensen JM, Crespo‐Bellido M, Dewey KG. Variation in hemoglobin across the life cycle and between males and females. Annals of the New York Academy of Sciences. 2019;1450(1):105–25. doi: 10.1111/nyas.14096 31074518

[36] Getaneh Z, Enawgaw B, Engidaye G, Seyoum M, Berhane M, Abebe Z, et al. Prevalence of anemia and associated factors among school children in Gondar town public primary schools, northwest Ethiopia: A school-based cross-sectional study. PloS one. 2017;12(12):e0190151. doi: 10.1371/journal.pone.0190151 29284032 PMC5746225

[37] Kiani B, Fatima M, Amin NH, Hesami A. Comparing geospatial clustering methods to study spatial patterns of lung cancer rates in urban areas: A case study in Mashhad, Iran. GeoJournal. 2022:1–11.

[38] Obasohan PE, Walters SJ, Jacques R, Khatab K. A Scoping Review of the Risk Factors Associated with Anaemia among Children Under Five Years in Sub-Saharan African Countries. International Journal of Environmental Research and Public Health. 2020;17: 8829. doi: 10.3390/ijerph17238829 33261060 PMC7731158

[39] Coleman M, Coleman M, Mabuza AM, Kok G, Coetzee M, Durrheim DN. Using the SaTScan method to detect local malaria clusters for guiding malaria control programmes. Malaria journal. 2009 Dec;8:1–6. 19374738 10.1186/1475-2875-8-68 PMC2679049

[40] Chirenda J, Gwitira I, Warren RM, Sampson SL, Murwira A, Masimirembwa C, et al. Spatial distribution of Mycobacterium tuberculosis in metropolitan Harare, Zimbabwe. PloS one. 2020;15(4):e0231637. doi: 10.1371/journal.pone.0231637 32315335 PMC7173793

[41] Kim J, Lee M, Jung I. A comparison of spatial pattern detection methods for major cancer mortality in Korea. Asia Pacific Journal of Public Health. 2016;28(6):539–53. doi: 10.1177/1010539516657871 27390024

[42] Ejigu BA, Wencheko E, Berhane K. Spatial pattern and determinants of anaemia in Ethiopia. PloS one. 2018;13(5):e0197171. doi: 10.1371/journal.pone.0197171 29775472 PMC5959194

[43] Foote EM, Sullivan KM, Ruth LJ, Oremo J, Sadumah I, Williams TN, et al. Determinants of anemia among preschool children in rural, western Kenya. Am J Trop Med Hyg. 2013;88(4):757–64. doi: 10.4269/ajtmh.12-0560 23382166 PMC3617865

[44] Roberts DJ, Matthews G, Snow RW, Zewotir T, Sartorius B. Investigating the spatial variation and risk factors of childhood anaemia in four sub-Saharan African countries. BMC public health. 2020;20(1):1–10. 31996196 10.1186/s12889-020-8189-8 PMC6990548

[45] Zerdo Z, Bastiaens H, Anthierens S, Massebo F, Masne M, Biresaw G, et al. Prevalence and associated risk factors of asymptomatic malaria and anaemia among school-aged children in Dara Mallo and Uba Debretsehay districts: results from baseline cluster randomized trial. Malaria Journal. 2021;20(1):1–12. 34645464 10.1186/s12936-021-03937-2 PMC8513194

[46] Moradi S, Arghavani H, Issah A, Mohammadi H, Mirzaei K. Food insecurity and anaemia risk: a systematic review and meta-analysis. Public Health Nutrition. 2018;21: 3067–3079. doi: 10.1017/S1368980018001775 30021665 PMC10261003

[47] FEWS NET. [cited 6^th^ May 2023]. Available from: https://fews.net/.

[48] White NJ. Anaemia and malaria. Malaria Journal. 2018;17: 371. doi: 10.1186/s12936-018-2509-9 30340592 PMC6194647

[49] School based deworming programme treats over 5M children–MINISTRY OF HEALTH. [cited 5^th^ May 2023]. Available from: https://www.health.go.ke/school-based-deworming-programme-treats-over-5m-children/.

[50] Government of Kenya launches deworming programme for pupils. Siaya (Kenya) March 11, 2020 –MINISTRY OF HEALTH. [cited 5^th^ May 2023]. Available from: https://www.health.go.ke/government-of-kenya-launches-deworming-programme-for-pupils-siaya-kenya-march-11-2020/.

[51] Mwandawiro C, Okoyo C, Kihara J, Simiyu E, Kepha S, Campbell SJ, Freeman MC, Brooker SJ, Njenga SM. Results of a national school-based deworming programme on soil-transmitted helminths infections and schistosomiasis in Kenya: 2012–2017. Parasites & vectors. 2019 Dec;12(1):1–8. doi: 10.1186/s13071-019-3322-1 30732642 PMC6367841

[52] National Malaria Control Programme, Ministry of Health. Kenya Malaria Strategy 2019–2023.Nairobi, 2019.

[53] Hulett JL, Weiss RE, Bwibo NO, Galal OM, Drorbaugh N, Neumann CG. Animal source foods have a positive impact on the primary school test scores of Kenyan schoolchildren in a cluster-randomised, controlled feeding intervention trial. Br J Nutr. 2014;111: 875–886. doi: 10.1017/S0007114513003310 24168874

[54] Murphy SP, Gewa C, Liang L-J, Grillenberger M, Bwibo NO, Neumann CG. School snacks containing animal source foods improve dietary quality for children in rural Kenya. J Nutr. 2003;133: 3950S–3956S. doi: 10.1093/jn/133.11.3950S 14672295

[55] Neervoort F, von Rosenstiel I, Bongers K, Demetriades M, Shacola M, Wolffers I. Effect of a School Feeding Programme on Nutritional Status and Anaemia in an Urban Slum: A Preliminary Evaluation in Kenya. Journal of Tropical Pediatrics. 2013;59: 165–174. doi: 10.1093/tropej/fms070 23243080

[56] Kulldorff M, Huang L, Pickle L, Duczmal L. An elliptic spatial scan statistic. Stat Med. 2006;25(22):3929–43. doi: 10.1002/sim.2490 16435334

[57] Endris BS, Dinant GJ, Gebreyesus SH, Spigt M. Geospatial inequality of anaemia among children in Ethiopia. Geospat Health. 2021;16. doi: 10.4081/gh.2021.1036 34726035

[58] Huang L, Pickle LW, Das B. Evaluating spatial methods for investigating global clustering and cluster detection of cancer cases. Statistics in medicine. 2008;27(25):5111–42. doi: 10.1002/sim.3342 18712778 PMC2575694

[59] Song C, Kulldorff M. Power evaluation of disease clustering tests. International journal of health geographics. 2003;2(1):1–8. 14687424 10.1186/1476-072X-2-9 PMC333429

[60] Hotez PJ, Molyneux DH. Tropical anemia: one of Africa’s great killers and a rationale for linking malaria and neglected tropical disease control to achieve a common goal. PLoS Negl Trop Dis. 2008;2: e270. doi: 10.1371/journal.pntd.0000270 18665256 PMC2474697

