# Supplementary figures and images for: Spatial variation and clustering of anaemia prevalence in school-aged children in Western Kenya
Source: PLoS One. 2023 Nov 27;18(11):e0282382. doi: 10.1371/journal.pone.0282382 (PMC10681207; doi:10.1371/journal.pone.0282382)

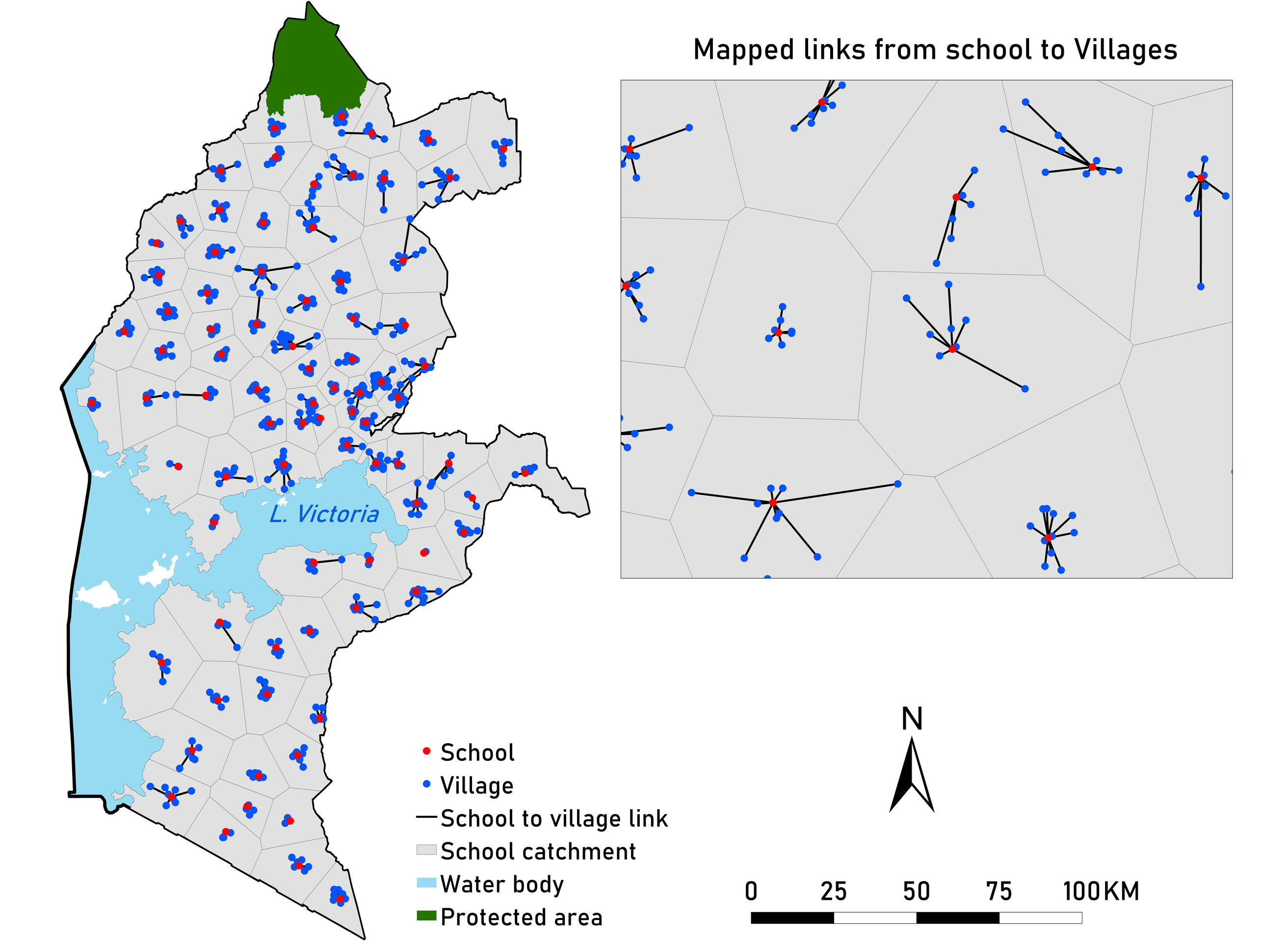

Supplement: S1 Fig — The Western Kenya shapefile was based on the County Integrated Development Plans 2021 [22]. (TIF) [file pone.0282382.s001.tif]

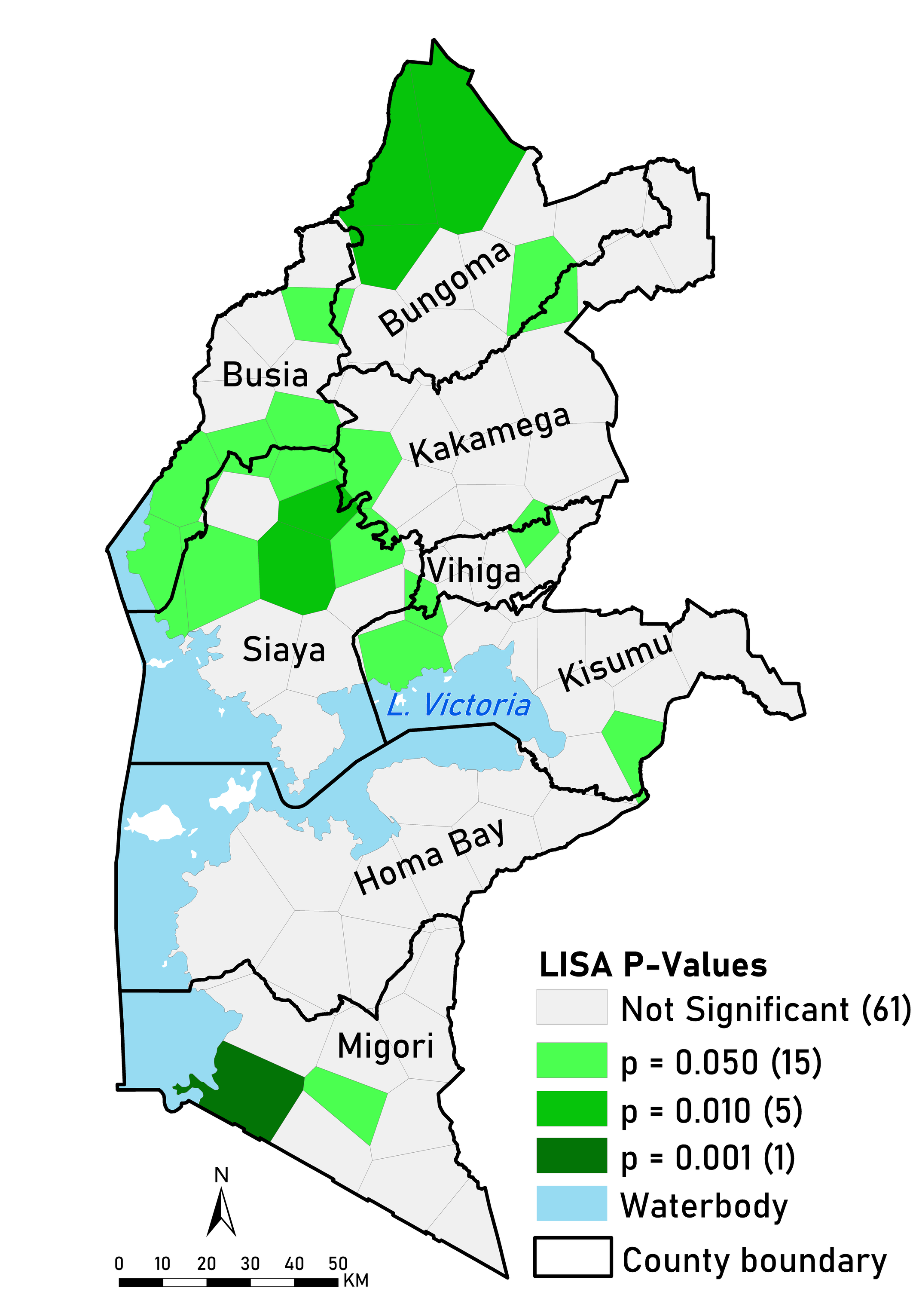

Supplement: S2 Fig — The Western Kenya county level shapefile was based on the County Integrated Development Plans 2021 [22]. (TIF) [file pone.0282382.s002.tif]
